# Predictive coding in psychopathology: mechanistic model or metaphorical re-description?

**DOI:** 10.3389/fnhum.2025.1743028

**Published:** 2026-01-16

**Authors:** Albandri Sultan Alotaibi

**Affiliations:** Department of Psychology, King Saud University, Riyadh, Saudi Arabia

**Keywords:** Autism spectrum disorder (ASD), hierarchical inference, mood or anxiety disorders, precision weighting, prediction error, predictive coding, Schizophrenia

## Abstract

Predictive coding (PC) has become a central framework in contemporary cognitive neuroscience, proposing that the brain operates as a hierarchical inference system that continuously minimizes the mismatch between predicted and actual sensory input. Its extension into clinical neuroscience has been accompanied by considerable enthusiasm, yet attempts to translate its computational principles into explanations of psychiatric and neurological disorders have yielded uneven results. The present review critically examines the clinical applicability of PC across three diagnostic domains: schizophrenia, autism spectrum disorder (ASD), and mood and anxiety disorders. Drawing on findings from neuroimaging, electrophysiology, and computational modeling, the discussion evaluates how disturbances in prediction error signaling, the precision weighting of sensory evidence relative to prior beliefs, and hierarchical inference have been proposed to relate to core clinical phenomena such as hallucinations, sensory hypersensitivity, and affective dysregulation. Particular attention is given to persistent theoretical tensions, including debates surrounding prior precision, the mapping between neural proxies and behavior, and the inconsistent use of PC terminology across diagnostic contexts. By adopting a structured and comparative approach, this review aims to clarify where predictive coding offers testable mechanistic insight into psychopathology, and where its explanatory scope remains limited or provisional.

## Introduction

The human brain is, in many ways, an indefatigable generator of expectations. It does not merely register the external world but continuously anticipates it, constructing hypotheses about the trajectory of a moving object, the tone of a voice, or even the likely intentions of another mind. The theory of predictive coding (PC) gives formal structure to this intuition. It proposes that the brain operates not as a passive sensory apparatus but as an active, inferential system, constantly testing and revising its hypotheses to minimize the discrepancy between prediction and input (Clark, 2013; Friston, 2005). Grounded in Bayesian logic and the minimization of free energy, this framework has, over the past two decades, acquired remarkable influence in cognitive and computational neuroscience. It offers a unifying grammar for understanding diverse mental functions, perception, motor control, even social cognition (Friston, 2010; Friston and Kiebel, 2009) though one might note that such breadth can be both its strength and its conceptual vulnerability.

Over roughly the last decade, predictive coding has extended its reach into clinical neuroscience, where its explanatory ambition has grown considerably. Researchers have begun to ask whether disturbances in the brain's inferential machinery could account for the complex symptom profiles of psychiatric and neurological disorders. Within this emerging view, hallucinations, sensory hypersensitivity, or affective blunting are no longer seen merely as isolated clinical features but as possible consequences of disrupted hierarchical inference (Adams et al., 2013; Sterzer et al., 2018). An overweighting of prior beliefs, for example, may not create outright resistance to counterevidence but can instead reduce effective learning rates and dampen the incorporation of new or immediate sensory input, a pattern that may contribute to the rigidity of delusional beliefs. Conversely, an under-weighting of priors can yield a perceptual world that feels unstable or fragmented, dominated by unpredictable sensory fluctuations. These interpretations are intuitively appealing, though it remains uncertain to what extent they describe genuine neurocomputational dysfunction rather than metaphorical re-descriptions of clinical phenomena.

Nevertheless, enthusiasm for predictive coding in psychiatry has been substantial. A number of reviews now celebrate its potential to integrate cognitive, neural, and phenomenological levels of explanation (Corlett et al., 2019; Friston et al., 2022). Yet, many of these contributions remain either overly abstract or limited to specific disorders. Meanwhile, recent work measuring prediction errors and precision weighting through concrete neural markers at the activity, laminar, and oscillatory levels (e.g., Vezoli et al., 2021) has begun to narrow the gap between computational constructs and their biological implementation. Building on these advances, the present review asks under which conditions such mappings remain mechanistic rather than merely descriptive. Does predictive coding explain psychotic and affective pathology through a common computational principle, or are we simply imposing a shared vocabulary upon distinct mechanisms? And perhaps most importantly, can a framework this elastic retain falsifiability, or does it risk explaining almost anything, and therefore nothing, too well?

The present review offers a contribution that complements, rather than replicates, previous overviews of predictive processing accounts in psychopathology. While existing reviews have summarized the growing use of predictive coding interpretations across schizophrenia, autism, and affective disorders, relatively little work has focused on the conceptual boundaries that clarify when predictive coding functions as a mechanistic model and when it risks becoming a broader descriptive metaphor. To address this gap, the current review outlines a comparative framework for understanding different computational “failure modes” (e.g., overprecise priors, attenuated prediction errors, hierarchical imbalance, impaired volatility learning) and considers how these patterns may manifest differently across diagnostic categories. By organizing these phenomena across hierarchical levels of inference (sensory, associative, and executive), the review aims to provide a clearer mapping between computational constructs and disorder-specific disruptions. A second contribution is to outline early conditions that show when predictive coding explanations become less convincing rather than more expansive, helping to keep the framework conceptually disciplined.

## Methodological approach

Although this review does not claim the status of a systematic review or meta-analysis, the methodological approach was designed to maintain transparency while allowing for the conceptual flexibility inherent to narrative synthesis. The primary objective was not merely to catalog findings, but to trace how predictive coding has been theoretically elaborated and empirically operationalized within clinical neuroscience.

A broad scoping search was conducted across PubMed, PsycINFO, and Google Scholar, covering publications from 2010 through early 2025. Search terms combined key concepts “*predictive coding, precision weighting, prediction error, hierarchical inference*” with disorder-specific terms relevant to schizophrenia, autism spectrum disorder (ASD), and mood or anxiety disorders.

From the initial body of literature, studies were purposefully selected based on several criteria. Eligible work was required to engage with predictive coding at a mechanistic level, offering substantive theoretical development or empirical evidence concerning precision weighting, prediction error propagation, or hierarchical inference. Priority was given to peer reviewed studies employing neurophysiological or computational techniques such as EEG, MEG, fMRI, and dynamic causal modeling (DCM). Works focusing exclusively on sensory prediction without clinical relevance, as well as non-peer reviewed materials, were excluded except where they provided essential theoretical context.

The inclusion strategy sought to balance breadth with coherence, emphasizing conceptual clarity rather than exhaustive coverage. Although narrative in form, the review adheres to principles of methodological transparency by explicitly outlining the search scope, inclusion criteria, and interpretive foundations guiding the synthesis. The resulting analysis should therefore be understood as an integrative, theoretically oriented evaluation rather than a statistical aggregation.

## The predictive coding framework: a primer

At its most basic level, predictive coding proposes that perception is not a passive reception of the world but an active inferential process. The brain is constantly generating hypotheses about the likely causes of its sensory inputs and updating these hypotheses considering new evidence (Clark, 2013; Friston, 2005). This ongoing dialogue between expectation and evidence is thought to occur within a hierarchically organized cortical architecture, in which higher levels encode abstract, slowly changing representations, and lower levels register immediate sensory details. Predictions flow downward, while prediction errors signal reflecting mismatches between expectation and input, travel upward through the hierarchy to revise the model. The process is continuous, recursive, and fundamentally conservative: the brain strives not to discover novelty for its own sake, but to minimize surprise.

Two concepts, in particular, have proved central to predictive coding explanatory appeal: precision weighting and hierarchical inference. Precision weighting refers to the system's dynamic estimation of the reliability- or expected precision- of a given signal (Feldman and Friston, 2010). When sensory input is noisy or ambiguous, the brain may down-weight its influence and rely more heavily on prior expectations; conversely, in an unpredictable environment, it must amplify the impact of sensory data. Neurophysiologically, this adaptive “gain control” is often attributed to the modulation of synaptic efficacy by neuromodulators such as dopamine or acetylcholine, effectively tuning the confidence placed on prediction error units (Rao and Ballard, 1999).

The second principle- hierarchical inference- reflects the idea that the brain's generative model is layered across multiple temporal and spatial scales (Friston, 2010). Higher-order areas generate predictions about latent causes of sensory events, while lower levels encode the more transient features of experience, such as contours, tones, or motion. This architecture allows the brain to interpret the world simultaneously in terms of what is happening now and what tends to happen in general. Crucially, communication between these levels is bidirectional and continuous, producing a self-correcting loop of expectation and revision.

Despite its elegance, predictive coding is not without controversy. Some critics have argued that its explanatory scope is so vast as to approach unfalsifiability (Keller and Mrsic-Flogel, 2018), while others question whether its computational operations can be meaningfully implemented in biological circuitry. Furthermore, the expansion of predictive coding into “active inference” models, which frame action itself as a means of minimizing prediction error, has blurred conceptual boundaries rather than clarified them (Friston et al., 2010). Still, the recent integration of computational modeling with empirical neuroimaging has begun to narrow this gap. For instance, studies employing dynamic causal modeling have demonstrated that precision weighting can be experimentally modulated, observable as context-dependent changes in self-inhibition within cortical microcircuits (Lecaignard et al., 2022). Such advances mark a turning point: predictive coding is shifting from a metaphorical scaffold toward a framework capable of generating falsifiable, quantitatively testable hypotheses about brain function.

It is in this transition -from theoretical elegance to empirical tractability- that the future of predictive coding is likely to rest. The framework's success will depend less on its rhetorical coherence than on its ability to specify how prediction and error interact within identifiable neural systems, and when these dynamics fail in the context of psychopathology.

## Clinical applications of predictive coding

The principles of predictive coding have been invoked to explain a wide spectrum of neuropsychiatric phenomena, from perceptual disturbances to deficits in social cognition. The underlying proposition is deceptively simple: that many symptoms of mental disorder may reflect perturbations in the brain's inferential machinery, in how it predicts, evaluates, and corrects its own sensory evidence. In practice, however, the clinical application of predictive coding is far from straightforward. Different disorders appear to manifest different “failure modes” of predictive processing, and the same computational vocabulary (prediction error, precision, priors) is often stretched to accommodate contradictory findings. Still, the framework's appeal lies precisely in its capacity to bridge levels of explanation, linking cellular computation to subjective experience.

In the following sections, I examine three diagnostic domains where predictive coding has been most influential: schizophrenia, autism spectrum disorder (ASD), and mood and anxiety disorders. Each of these fields illustrate not only the promise but also the fragility of predictive coding as a clinical theory, its capacity to integrate diverse data, yet its tendency to overgeneralize.

### Schizophrenia: aberrant precision and the disintegration of belief

- **Failure mode:** Psychosis has been increasingly conceptualized as involving disturbances in precision weighting, such that the reliability of prediction errors is not consistently estimated across hierarchical levels. Depending on context, this can lead to low-level sensory fluctuations being treated as overly significant, or to an increased reliance on top-down priors, possibly as a compensatory response to unstable or noisy sensory input (Sterzer et al., 2018).
- **Hierarchical level:** Disruption appears most pronounced at higher, integrative levels of the cortical hierarchy, particularly within associative regions implicated in belief formation and contextual integration. Alterations at these levels are frequently accompanied by changes in lower sensory processing, which may reflect compensatory dynamics rather than primary dysfunction, and whose functional role likely depends on task demands and inferential context (Haarsma et al., 2022).
- **Behavioral predictions:** These computational mismatches have been linked to hallucinations, where perception becomes overly guided by expectations rather than by sensory input, and to delusional beliefs, which may arise as attempts to make sense of experiences that feel uncertain or noisy (Corlett et al., 2019; Powers et al., 2017). Related disturbances in agency have also been described, particularly when individuals have difficulty recognizing their own actions or thoughts as self-generated (Sterzer et al., 2018).
- **Neural/Computational evidence:** Many electrophysiological studies report reduced mismatch negativity (MMN) in psychosis, which is often taken as evidence of weaker prediction-error processing (Umbricht and Krljes, 2005). Neuroimaging findings also suggest that the anterior insula and dorsal anterior cingulate cortex respond less consistently to prediction violations, especially under conditions of uncertainty (Sterzer et al., 2018).
- **Unresolved tensions:** An important unresolved issue concerns the direction of the disturbance. Some predictive-coding models propose that psychosis begins with overly precise sensory prediction errors, while others suggest that weak or unreliable higher-level priors play a more central role (Sterzer et al., 2018). This disagreement makes it difficult to determine where, within the hierarchical system, the disturbance first arises.

### Autism spectrum disorder: inflexible or over-precise prediction errors?

- **Failure mode:** Autism spectrum disorder (ASD) has often been described in predictive-coding terms as involving unusually precise low-level prediction errors, such that small sensory changes are treated as meaningful and demand attention. This pattern may limit the formation of more stable, generalized top-down expectations, leading perception to remain closely tied to immediate sensory input (Pellicano and Burr, 2012; Lawson et al., 2017).
- **Hierarchical level:** The disruption appears to involve the transition from sensory to associative levels of processing. When sensory prediction errors are assigned unusually high precision, incoming sensory signals may dominate processing, making it more difficult for higher-level systems to distinguish between meaningful changes in the environment and minor or irrelevant variations (Pellicano and Burr, 2012; Lawson et al., 2017).
- **Behavioral predictions:** At the behavioral level, this pattern has been linked to sensory hypersensitivity, where incoming sensory input is experienced as overly intense, and to an insistence on sameness, which may reflect an effort to reduce uncertainty in an environment that is perceived as unpredictable (Pellicano and Burr, 2012; Lawson et al., 2017).
- **Neural/Computational evidence:** Magnetoencephalography studies report reduced beta-band activity during prediction tasks, a pattern often interpreted as reflecting weaker top-down predictive influence (Lawson et al., 2017). Computational modeling studies further suggest atypical learning under changing conditions, with individuals showing difficulty adjusting their prior expectations when environmental contingencies change (Lawson et al., 2017; Lieder et al., 2019).
- **Unresolved tensions:** An important open question concerns whether atypical priors in autism are best understood as weak, in the sense that they provide limited constraint on sensory input, or as inflexible, in that they are slow to update when conditions change. Although both accounts can explain behavioral rigidity, they point to different underlying computational mechanisms (Cannon et al., 2021).

### Mood and anxiety disorders: affective inference and biased priors

- **Failure mode:** Mood and anxiety disorders are often described as involving disturbances in affective inference, where the brain assigns maladaptive weight to internal feeling states or signals related to threat. In depression, this pattern is commonly reflected in reduced sensitivity to positive prediction errors, limiting the updating of positive expectations (Rutledge et al., 2017; Gilbert et al., 2022). In anxiety, difficulties are more often linked to heightened weighting of uncertainty or threat-related signals, leading to persistent expectations of negative outcomes (Browning et al., 2015; Nelson et al., 2015).
- **Hierarchical level:** Disruption in mood and anxiety disorders is most often discussed at associative and executive levels of processing. These levels include circuits involving the ventral striatum and the medial prefrontal cortex, which play central roles in reward evaluation and the regulation of emotional responses (Rutledge et al., 2017; Gilbert et al., 2022).
- **Behavioral predictions:** In depression, reduced sensitivity to reward-related prediction errors has been linked to anhedonia and the persistence of negative or pessimistic expectations (Gilbert et al., 2022; Rutledge et al., 2017). In anxiety, behavior is more often shaped by strong expectations of harm, which can contribute to intolerance of uncertainty and to continued anticipation of threat even in situations that are objectively safe (Browning et al., 2015; Nelson et al., 2015).
- **Neural/Computational evidence:** Research on major depressive disorder has consistently reported reduced reward-related prediction error signals in the ventral striatum, a finding linked to diminished responsiveness to positive outcomes (Rutledge et al., 2017). In anxiety disorders, neurophysiological studies have identified altered oscillatory activity in the anterior cingulate cortex during threat anticipation, suggesting difficulties in distinguishing between threat and safety signals under conditions of uncertainty (Hein et al., 2022).
- **Unresolved tensions:** A key unresolved issue concerns how subjective emotional experience maps onto computational variables such as gain or precision. At present, this relationship remains only partly specified, making it difficult to determine whether these models capture the underlying causes of affective distress or mainly describe its downstream consequences (Gilbert et al., 2022).

## Transdiagnostic reflections

Across schizophrenia, ASD, and mood and anxiety disorders, predictive coding has been proposed as a common explanatory framework. At the same time, its terminology is sometimes used in a broad way that risks obscuring important differences between conditions. Although the disorder-specific sections above describe distinct patterns of difficulty, several transdiagnostic issues emerge that continue to pose challenges for the field. Recent neurophysiological evidence further supports this continuum-based perspective, showing that individuals can be differentiated along autism–schizophrenia dimensions by the neural strategies they employ during predictive processing rather than by categorical diagnosis alone (Tarasi et al., 2023). Such findings suggest that hierarchical imbalances in predictive coding may vary parametrically across conditions, reinforcing the need for transdiagnostic models that capture both shared mechanisms and disorder-specific expressions.

- **Precision weighting as a shared issue:** Disturbances in how precision is assigned appear across diagnostic categories and may represent a common computational theme. However, the clinical expression of these disturbances varies depending on the level of the hierarchy at which they occur. As summarized in Table 1, imbalances at early sensory levels are more often discussed in relation to autism, where perception remains strongly tied to immediate input, whereas disruptions at higher associative levels are more frequently emphasized in psychosis, where sensory information may be interpreted in unusual or idiosyncratic ways.
- **Difficulties with volatility estimation:** A further shared feature across anxiety and autism spectrum disorder concerns difficulties in estimating environmental volatility. When changes in the environment are hard to distinguish from random fluctuations, behavior may become poorly calibrated. In anxiety, this is often expressed as heightened vigilance and persistent monitoring for threat, whereas in autism it more commonly appears as behavioral rigidity and a preference for stable routines.
- **Convergence and divergence across disorders:** Predictive coding has been useful in linking seemingly different phenomena, such as delusions and sensory hypersensitivity, through common computational principles. At the same time, it remains necessary to clarify when such disturbances lead to disorder-specific patterns and when they reflect more general vulnerabilities shared across conditions.

**Table 1:** Comparative taxonomy of computational failure modes.

**Computational failure mode**	**Hierarchical Level**	**Direction of disruption**	**Behavioral manifestation**	**Representative evidence**	**Associated disorder**
Overprecise priors	Associative → Executive	↑ Precision of prior beliefs	Rigid belief updating; resistance to contradictory evidence	Powers et al., 2017; Corlett et al., 2019	Schizophrenia (delusional ideation)
Weak / underweighted priors	Sensory	↓ Prior influence on perception	Heightened sensory responsiveness; reduced contextual modulation	Lieder et al., 2019; Goris et al., 2022; Palmer et al., 2017	Autism Spectrum Disorder
Underweighted prediction errors	Associative	↓ PE influence	Slow updating under volatility; reduced learning rates	Ramos-Grille et al., 2022; Haarsma et al., 2022	Depression / anhedonia
Overweighted prediction errors	Sensory → Associative	↑ Unsigned PE	Noisy evidence accumulation; aberrant salience	Haarsma et al., 2021; Roiser et al., 2009	Psychosis-spectrum
Hierarchical imbalance	Multilevel (sensory executive)	Dysregulated precision across levels	Confusion between expected and unexpected inputs; impaired volatility inference	Sapey-Triomphe et al., 2023	Autism–Schizophrenia continuum
Impaired volatility estimation	Executive	Misestimation of environmental change	Maladaptive switching; unstable priors	Lawson et al., 2017; Nassar et al., 2010	Anxiety disorders

See Box 1 for operational definitions of these failure modes.

Box 1 **Computational glossary of failure modes.**

To reduce conceptual overlap among the failure modes listed in Table 1, the key terms are defined below in operational and computational terms. These definitions are intended to clarify how each failure mode is used in the present review.

• **Weak or underweighted priors:** A state in which low precision is assigned to top-down expectations. As a result, perception and belief formation rely heavily on incoming sensory input, with limited integration across experiences.

• **Overprecise or rigid priors:** A state in which excessive precision is assigned to prior expectations. In this case, beliefs become resistant to updating, even when incoming evidence contradicts existing expectations.

• **Hierarchical imbalance:** A mismatch in precision assignment across hierarchical levels, such as relatively high precision at sensory levels combined with lower precision at higher associative levels. This imbalance can reduce the ability to distinguish between expected and unexpected inputs.

• **Impaired volatility estimation:** A difficulty in estimating the rate or likelihood of change in the environment, typically at higher or executive levels of processing. This can lead to inappropriate switching between beliefs or strategies, or to reduced learning when environmental contingencies change.

Addressing these issues requires moving beyond the use of predictive coding as a broad descriptive framework toward more explicit and testable mechanistic accounts. By relating transdiagnostic claims to the falsifiability criteria outlined in the Discussion, the framework can be better positioned as a cautious but informative tool for clinical research.

## Discussion: synthesis and future directions

The present review points to a field that is conceptually rich but still methodologically unsettled. Predictive coding has become an influential framework for understanding psychopathology, offering a shared language across perception, learning, and affect. At the same time, its rapid expansion across multiple diagnostic categories has sometimes reduced conceptual clarity. Rather than viewing the field as moving between opposing positions, it may be more accurate to see a productive division of effort, in which tightly controlled mechanistic studies and broader integrative approaches develop in parallel, each addressing different questions about predictive processing (Sterzer et al., 2018).

### Worked empirical tensions in predictive coding accounts

Although predictive coding provides a flexible explanatory framework, its empirical value ultimately depends on whether specific, testable predictions are supported by data. In this section, three classes of empirical tension are used to highlight current boundary conditions of predictive-coding models, particularly where theoretical expectations do not map cleanly onto observed behavior or neural signals.

**1. Mismatch negativity (MMN) and belief updating**

Within predictive-coding accounts, mismatch negativity is commonly interpreted as a neural marker of sensory prediction error. On this view, reduced MMN amplitude is taken to indicate weakened error signaling and, by extension, reduced belief updating. In schizophrenia, however, this mapping has proven difficult to sustain. While reductions in MMN are robust and widely replicated (Umbricht and Krljes, 2005), corresponding impairments in behavioral learning and updating are not consistently observed.

For example, Erickson et al. (2016) report attenuated MMN responses in auditory oddball paradigms, yet these neural differences do not reliably predict learning rates or adaptive updating in tasks that directly manipulate statistical regularities. Similar dissociations have been noted in other studies, where preserved behavioral adjustment coexists with reduced MMN amplitude (Haigh et al., 2017). Together, these findings suggest that MMN may primarily reflect early sensory mismatch detection rather than the higher-level inferential processes required for belief revision. In this sense, prediction-error signals indexed by MMN may be attenuated or filtered before they meaningfully influence the stages of processing that guide behavior.

**2. Prediction-error magnitude and updating under volatility**

Hierarchical Bayesian models generally assume that larger unsigned prediction errors should lead to stronger belief updating, especially in environments characterized by high volatility. Empirical findings, however, suggest that this relationship is not always straightforward. In volatile contexts, individuals may show strong prediction-error signals without corresponding changes in their belief estimates over time.

For example, Browning et al. (2015) report that anxious individuals display heightened prediction-error responses when outcomes are uncertain and aversive, yet their beliefs remain relatively inflexible and do not adjust in line with changing contingencies. This pattern indicates that the size of a prediction error alone may be insufficient to drive adaptive updating. Instead, belief revision appears to depend on higher-order inferences about uncertainty and volatility, which may themselves be altered in anxiety.

**3. Oscillatory markers and precision weighting**

Predictive-coding models often link beta-band oscillatory activity to top-down predictions and the weighting of precision, leading to the expectation that beta activity should vary systematically with certainty. Empirical support for this mapping, however, has been mixed. Studies examining laminar organization and oscillatory dynamics have identified situations in which beta-band activity does not reliably track experimentally manipulated uncertainty, or does so in a manner that diverges from model predictions (Bastos et al., 2020).

When proposed neural markers of precision fail to covary with expected uncertainty, the link between computational constructs and specific neurophysiological signals becomes less clear. These findings suggest that beta-band activity may reflect multiple processes, rather than serving as a direct or exclusive marker of precision weighting.

Taken together, these examples show that empirical tensions in predictive coding often arise from systematic dissociations between neural proxies and behavioral outcomes. Such findings do not undermine Bayesian inference as a general framework, but they do highlight limits in current assumptions about neural implementation. As summarized in Table 1, many of these tensions can be traced to distinct patterns of disruption across hierarchical levels, emphasizing the need for more precise mappings between theory, task, and neural measures.

As shown in Table 1, computational failure modes vary across hierarchical levels and disorders. Importantly, these failure modes are not intended as diagnostic signatures, but as testable points at which predictive coding explanations can succeed or fail.

### Falsifiability and mechanistic constraints

The dissociations discussed above provide a basis for thinking about falsifiability in predictive coding accounts. Predictive-coding explanations become less convincing under several conditions. First, problems arise when neural signals interpreted as prediction errors do not show a reliable relationship with behavioral updating in tasks that manipulate environmental volatility (Browning et al., 2015). Second, the framework is challenged if prior beliefs remain largely unchanged despite clear and sustained shifts in environmental statistics, suggesting limited sensitivity to new evidence. Third, difficulties emerge when neural markers proposed to reflect precision, such as specific oscillatory patterns, fail to vary in line with task-related uncertainty (Bastos et al., 2020). Finally, if alternative models, such as reinforcement-learning approaches, aberrant salience accounts, or cognitive-control models, consistently offer more parsimonious and testable explanations of the same data, the added explanatory value of a hierarchical generative framework becomes less clear.

Importantly, these criteria are not intended to reject predictive coding as a whole. Rather, they help distinguish between core Bayesian claims about inference and learning, and more specific implementational assumptions concerning how these processes are measured at the neural level. Clarifying this distinction is essential for evaluating which aspects of the framework are being tested, and which remain provisional.

### From aspiration to phenotyping

Computational phenotyping within predictive-coding frameworks should no longer be viewed as purely aspirational. A growing body of work has begun to operationalize individual differences using hierarchical Bayesian models that estimate parameters such as prior precision, learning rates, and sensitivity to uncertainty across conditions including psychosis, ASD, and mood disorders (Powers et al., 2017; Lawson et al., 2017; Rutledge et al., 2017). These approaches demonstrate that key theoretical constructs can be quantified at the level of individual participants.

At the same time, the translation of such parameters into routine clinical decision-making remains limited. Using computational estimates to guide treatment selection, predict outcomes, or tailor interventions continue to represent a major goal rather than an established practice. Addressing this gap will require closer integration between computational modeling, longitudinal clinical data, and intervention studies.

### Comparative advantages: when predictive coding is useful, and when other models are more informative

A constrained critique of predictive coding requires specifying the situations in which it offers clear advantages, as well as those in which alternative models provide more precise or testable accounts.

- **Cases where other models are more informative:** Reinforcement-learning models often provide more detailed and algorithmically explicit accounts of trial-by-trial learning in reward-based tasks. In studies of depression, parameters derived from Q-learning models have been shown to predict specific impairments in reward learning more directly than broader predictive coding constructs such as altered precision weighting (Huys et al., 2015; Rutledge et al., 2017). In such contexts, reinforcement-learning approaches may offer clearer predictions and more straightforward links between behavior and model parameters.
- **Cases where predictive coding offers an advantage:** Predictive coding is particularly useful when the goal is to relate processes across multiple levels of description. Its hierarchical, generative structure allows low-level sensory differences and higher-level behavioral patterns to be considered within a single framework. As summarized in Table 1, this approach makes it possible to relate phenomena such as sensory hypersensitivity in autism to broader patterns of behavioral rigidity, through shared assumptions about precision assignment across levels of the hierarchy (Pellicano and Burr, 2012; Lawson et al., 2017). This form of cross-level integration is less readily captured by reinforcement-learning or sensory-gating models, which typically focus on more restricted aspects of processing.

## Conclusion

Predictive coding continues to offer a valuable framework for relating perception, learning, and affect across psychiatric conditions. At the same time, the analyses presented here indicate that its explanatory power depends on careful attention to empirical constraints and levels of explanation. By examining concrete dissociations between neural signals, computational assumptions, and behavior, this review highlight where current mappings remain uncertain and where alternative models provide clearer accounts. Rather than weakening the framework, such limitations help define the conditions under which predictive coding can be meaningfully tested and refined. Progress in this area will depend less on expanding the scope of the framework, and more on specifying its commitments, its points of failure, and its role alongside neighboring models in clinical science.
